# Quantification of patellar tendon strain and opportunities for personalized tendon loading during back squats

**DOI:** 10.1038/s41598-023-35441-9

**Published:** 2023-05-29

**Authors:** K. Weidlich, F. Mersmann, T. Domroes, A. Schroll, S. Bohm, A. Arampatzis

**Affiliations:** Department of Training and Movement Sciences, Humboldt-Universität zu Berlin, Philippstr. 13, Haus 11, 10115 Berlin, Germany

**Keywords:** Biophysics, Physiology

## Abstract

Tendon strain during exercise is a critical regulatory factor in tendon adaptive responses and there are indications for an optimal range of strain that promotes tendon adaptation. Back squats are used to improve patellar tendon properties in sport and clinical settings. To date, the operating patellar tendon strain during back squats is unknown and current recommendations for individual exercise loading are based on the one repetition maximum (1RM). Here, we quantified patellar tendon strain during loaded back squats at 40, 60 and 80% of the 1RM and during maximum isometric knee extension contractions (MVC) using ultrasonography. Kinematics, ground reaction forces and muscle electromyographic activity were also recorded. Additionally, maximum tendon strain during the MVC and the percentage of 1RM were used as explanatory variables to estimate the individual patellar tendon strain during the squats. Strain increased with increasing 1RM loading (4.7 to 8.2%), indicating that already medium-loading back squats may provide a sufficient stimulus for tendon adaptation. The individual variability was, however, too high to generalize these findings. Yet, there was a high agreement between the individually estimated and measured patellar tendon strain (R^2^ = 0.858) during back squats. We argue that this approach may provide new opportunities for personalized tendon exercise.

## Introduction

There is evidence that the magnitude of tendon strain under load is a critical mechanical factor for the regulation of the anabolic and catabolic responses of tendon fibroblasts^1,2^ as it affects the expression of several growth factors, the synthesis of matrix proteins and collagen type I production^3,4^. The concept of the ‘homeostatic calibration point’^5,6^ proposes the existence of an upper and lower limit in the applied tissue strain to induce a homeostatic perturbation of the internal cellular tension that regulates catabolic and anabolic responses. In fact, experimental studies indicate that a certain range of tendon strain may induce a net anabolic stimulation^7,8^, which led to the idea of a ‘sweet spot’ of strain to promote the tendon’s mechanical and morphological properties^9,10^. Mechanical loading that induces strain magnitudes close to the ‘sweet spot’ would stimulate cells for matrix production, whereas too high or too low strains will lead to matrix deterioration^7,9^. In a series of systematic intervention studies, we found that cyclic loading of the tendon with strain values between 4.5 and 6.5% was an effective mechanical stimulus to increase tendon stiffness, tendon elastic modulus and trigger tendon hypertrophy^11–13^. Therefore, to improve the efficacy of tendon adaptation in both healthy and pathological conditions, knowledge about the achieved tendon strain during certain exercise loading is needed^14^.

In sports and rehabilitative training settings, the free-weight barbell back squat is a fundamental full-body exercise and is particularly performed to improve both knee extensor muscle strength^15,16^ and patellar tendon properties^17,18^. Several studies investigating the resulting knee joint moment^19,20^ and the electromyographic activity (EMG) of the knee extensors^21,22^ during squats observed a gradual increase in these parameters as a function of external load. Due to the gradual increase in the resultant knee joint moments and EMG-activity of the knee extensor muscles the magnitude of the mechanical loading for both muscle and tendon adaptation is usually regulated by the percentage of the one repetition maximum (1RM) in training and clinical practice^23^. However, the actual patellar tendon strain achieved during back squats and the influence of external load is currently unknown.

The force applied to the patellar tendon and the normalized patellar tendon stiffness (i.e., slope of the tendon force-strain curve) are the two main parameters that affect patellar tendon strain during back squats. Individual variability in maximum muscle strength and normalized tendon stiffness may result in different magnitudes of tendon strain between individuals at the same percentage of the 1RM, which complicates the regulation of tendon loading based on the 1RM. In fact, although there is generally a strong association between muscle strength of the knee extensors and normalized patellar tendon stiffness, the individual variability of this relationship is high^24^. There is a large number of studies that reported imbalances between knee extensor muscle strength and normalized patellar tendon stiffness at the individual level^25–27^. This variable relationship between muscle strength and normalized tendon stiffness suggests that training recommendations for tendon loading based on the 1RM^23^ do not control for the actual tendon strain under load and may promote different metabolic responses between individuals that train at a given 1RM.

Earlier studies reported a relationship between the knee extension moment during a maximum voluntary isometric contraction (MVC) and the 1RM load during squats^28,29^. Due to this association, we can expect a relationship between the individual tendon strain achieved during an MVC and during the percentages of the 1RM in loaded squats. Assuming the above-mentioned relationship, it would be possible to estimate individual patellar tendon strain during loaded back squats using the achieved patellar tendon strain during an MVC and the 1RM percentages as independent variables. The quantification of patellar tendon strain during a maximum voluntary isometric knee extension contraction is established as diagnostic tool in training and clinical research^25,30^ and its application is less challenging compared to patellar tendon strain measurements during loaded back squats. A prediction of the individual patellar tendon strain during loaded back squats using easily measurable explanatory variables may introduce new possibilities for individualized tendon loading in clinical and sports practice.

The purpose of this study was (a) to quantify patellar tendon strain during back squats at different percentages of the 1RM and (b) to estimate the individual patellar tendon strain with easily measurable explanatory variables. We hypothesized a high individual variability of the achieved patellar tendon strain during back squats at the same percentages of the 1RM. Furthermore, we assumed that the percentage of the 1RM during the squat and patellar tendon strain during an isometric MVC of the knee extensors can be used as explanatory variables to estimate individual patellar tendon strain during back squats.

## Methods

### Participants and experimental design

Thirteen active athletes with various sports background (i.e., basketball, gymnastics, track and field, soccer and weightlifting) with at least two years of strength training experience who have executed squats with additional weight at least once per week in the past year were recruited for the present study. Three of them were excluded because of insufficient data quality of the tendon ultrasound images during the squats due to probe motion artifacts and, additionally, in one participant due to ultrasound image artifacts that occurred during the isometric contractions. In the remaining ten participants (5 males, 5 females, 30.5 ± 3.5 years, 172.6 ± 11.7 cm, 73.6 ± 15.5 kg) patellar tendon strain, ground reaction forces (GRF), kinematics and EMG-activity of the rectus femoris (RF), vastus lateralis (VL), vastus medialis (VM), and biceps femoris (BF) were measured during loaded back squats at 40%, 60% and 80% of the 1RM. Further, the patellar tendon force–elongation relationship and maximum strain were determined for an isometric MVC of the knee extensors. All measured parameters were obtained from the dominant leg, which was defined as the one used to kick a ball. The measurements during the loaded squats and the MVC were taken in a randomized order on two different days with a minimum interval of one week in between to ensure complete regeneration. The participants were instructed to refrain from vigorous physical activity 48 h before the measurements. The study was approved by the ethics committee of the Humboldt-Universität zu Berlin (reference number HU-KSBF-EK_2020_0022), followed the standards of the Declaration of Helsinki and the participants provided their written informed consent to the experimental procedure.

### Measurement of patellar tendon elongation during loaded back squats

After an individual warm-up, the 1RM was determined for a squat with the reversal point at about 60° knee angle (0° full extension) for each participant. The reversal point of 60° knee angle was chosen because it is close to the optimal position for knee extensor muscle force exertion considering its force–length relationship^31^. Since the 1RM load for this range of motion was unknown for most of the participants, the maximum vertical ground reaction forces during a maximum isometric squat trial at 60° knee angle against a fixed barbell served as a first orientation for the subsequent assessment of the 1RM^32^. The 1RM was then tested under dynamic conditions and identified after 2.00 ± 0.71 attempts. The squat rack was adjusted in height so that the participants began the squat at approximately 65° of knee flexion. This starting position proved to be favorable for the adjustment of the ultrasound probe, which will be described in more detail below. In each trial, the participants lifted the weight from the starting position to upright standing, performed one squat to 60° knee flexion and finished the squat with full extension. The participants were instructed to perform the movement with a constant angular velocity of about 10°/s, which was monitored via the angular change of the knee joint angle in the kinematic data and guided by verbal assistance. The speed of movement during the squat was adjusted to represent a loading profile that is considered particularly effective for adaptive response of the tendon, which includes a strain duration of about 3 s per loading cycle in the suggested optimal range magnitude^11,12^.

**Figure 1:**
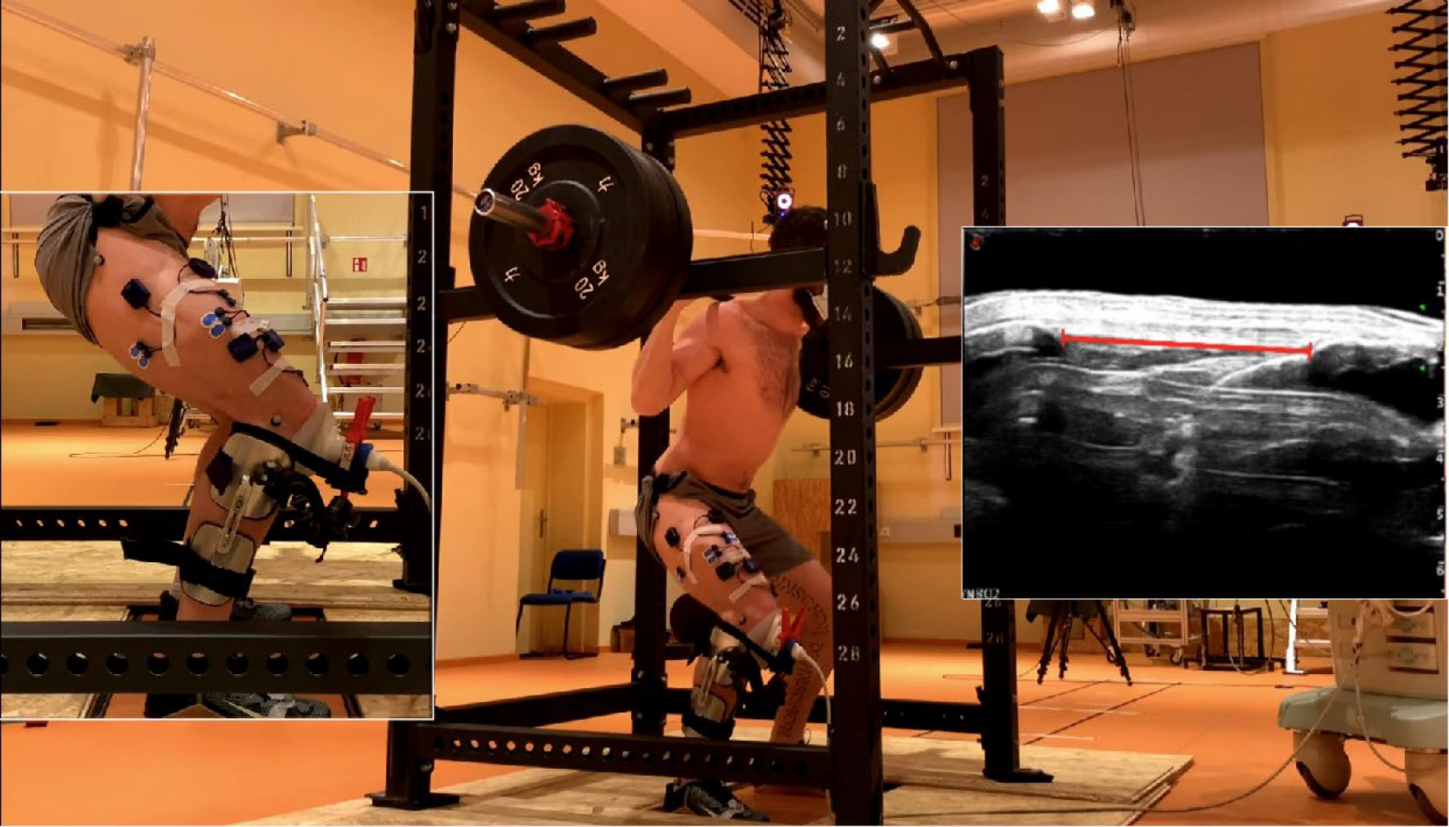
Middle: Experimental setup for determining patellar tendon elongation during the loaded squats. Each foot was placed on a force plate to measure the ground reaction forces of the two legs separately. Left: A 10-cm ultrasound probe was placed over the patellar tendon to measure tendon elongation. Reflective markers were used to determine joint kinematics, and surface electromyography of vastus lateralis, vastus medialis, rectus femoris and biceps femoris was used to examine muscle activation patterns. Right: Patellar tendon elongation was measured by digitizing the displacement of its insertion points at the caudal patella and the tibial tuberosity (red line).

A 10-cm ultrasound probe (MyLab60; Esaote, Genoa, Italy; probe: LA923, 7.5 MHz; 25 Hz image frequency) was placed perpendicular to the skin and aligned with the longitudinal axis of the patellar tendon with a modified knee brace (Fig. 1). Subsequently, three squat trials each were captured with 80%, then 60% and finally 40% of the 1RM. The length of the patellar tendon during the dynamic squats was measured by tracking the displacement of its attachment points at the tibial tuberosity and the caudal pole of the patella using a semi-automated tracking software (Tracker Video Analysis and Modeling Tool V. 5.5, Open Source Physics, Aptos, California, USA) and a moving average filter (windows size 0.24 s) was applied. From the measured patellar tendon length, we subtracted the tendon rest length to determine tendon elongation during the squats. Patellar tendon rest length was measured in a seated position at 60° knee joint angle. The foot was rested on a height-adjustable stool and the participants were requested to completely relax their knee extensors and flexors. The slackness of the tendon was accounted for using a spline fit through the deep insertion marks and two additional points along the border of the slack tendon^26^. Tendon strain was calculated by dividing the measured elongation by the rest length. The analysis of patellar tendon elongation and strain was confined to the last 10° of knee flexion and the first 10° of knee extension relative to the movement reversal point to minimize potential errors due to probe displacements. The duration of the analyzed sequence was 1.99 ± 0.43 s.

### Kinetics and EMG-activity during loaded back squats

The resultant ankle, knee and hip joint moments of the dominant leg during the dynamic loaded back squats were calculated using inverse dynamics^33^.

$${\mathrm\it{M}}_{\mathrm\it{j}}= - {\mathrm\it{M}}_{\mathrm\it{F}}-\left({\mathrm\it{r}}_{\mathrm\it{j}}\times \mathrm\it{F}\right)- \sum_{\mathrm\it{i}=1}^{\mathrm\it{n}}\left({\mathrm\it{r}}_{\mathrm\it{ji}}\times {\mathrm\it{G}}_{\mathrm\it{i}}\right)+ \sum_{\mathrm\it{i}=1}^{\mathrm\it{n}}\left({\mathrm\it{r}}_{\mathrm\it{ji}}\times {\dot{\mathrm\it{p}}}_{\mathrm\it{i}}\right)+ \sum_{\mathrm\it{i}=1}^{\mathrm\it{n}}{\dot{\mathrm\it{H}}}_{\mathrm\it{i}}$$

where ${\mathrm\it{M}}_{\mathrm\it{j}}$ is the $\mathrm\it{j}$th joint moment, ${\mathrm\it{M}}_{\mathrm\it{F}}$ the frictional moment, $\mathrm\it{F}$ the ground reaction force, ${\mathrm\it{r}}_{\mathrm\it{j}}$ the position vector between the $\mathrm\it{j}$th joint and the point of force application, ${\mathrm\it{r}}_{\mathrm\it{ji}}$ the position vector between the $\mathrm\it{j}$th joint and centre of mass of the $\mathrm\it{i}$th segment, ${\mathrm\it{G}}_{\mathrm\it{i}}$ the force of gravity on the $\mathrm\it{i}$th segment, ${\dot{\mathrm\it{p}}}_{\mathrm\it{i}}$ the first derivative of the $\mathrm\it{i}$th segment impulse, ${\dot{\mathrm\it{H}}}_{\mathrm\it{i}}$ the first derivative of the $\mathrm\it{i}$th segment angular momentum and $\mathrm\it{n}$th the number of segments. Reflective markers were placed at the following anatomical reference points: 7th thoracic vertebra, greater trochanter, condylus lateralis femoris, condylus medialis femoris, the medial and lateral malleolus, and over the second metatarsal bone. The markers were captured with ten cameras (5 × T20, 5 × T20-S) of a Vicon motion capture system (version 1.8.5, Vicon Motion Systems, Oxford, UK) at 250 Hz and simultaneously the GRF of both legs was recorded using two force plates (40 x 60 cm, AMTI BP400600-200, Advanced Mechanical Technology, Inc. Watertown, MA, USA) at a sampling frequency of 1 kHz. The kinematic and GRF data were filtered using a fourth-order low-pass zero-phase shift Butterworth filter with a 20 Hz cut-off frequency. Joint moments and angles were calculated in the sagittal plane using the horizontal and vertical coordinates of the digitized anatomical reference points. To identify potential changes in the technical execution of the squats, the distribution of the generated GRF between legs was investigated, and we calculated the ratio of the resultant hip to knee joint moment and the ratio of resultant ankle to knee joint moment in each loading percentage of the 1RM. As additional parameter for the body position, the trunk angle to the horizontal plane (90° defined as upright) was calculated.

The EMG-activity of RF, VL, VM and BF of the dominant leg was measured during the squats using a 16-channel wireless EMG system (Myon m320; Myon AG, Schwarzenberg, Switzerland), with a sampling frequency of 1 kHz. Two bipolar EMG electrodes (Blue Sensor N, Ambu GmbH, Bad Nauheim, Germany, 2 cm, 0.8 cm^2^ surface area) were placed on each muscle belly according to the Surface ElectroMyoGraphy for Noninvasive Muscle Assessment of Muscles (SENIAM) after appropriate preparation of the skin^34^. A second-order high-pass zero-phase shift Butterworth filter with a 6 Hz cut-off frequency, a full-wave rectification and a low-pass zero-phase filter with a 3 Hz cut-off frequency were applied to the raw EMG data. EMG-activity was evaluated over the selected range of motion and normalized to the EMG-activity during a separate single leg isometric maximal knee extension at 60° knee joint angle for RF, VL, VM, and knee flexion at 30° joint angle for BF, respectively, with the average calculated in a 0.2 s time window around the achieved maximum.

### Assessment of the patellar tendon force–elongation relationship and maximum strain

After a standardized warm-up including ten submaximal isometric knee extension contractions, the participants performed two MVCs at a knee angle of 60° and a hip angle of 85° (0° full extension) on a dynamometer (Biodex Medical, Shirley, New York, USA). The higher value of the two MVCs was considered the maximum knee extension moment. Then, the 10-cm probe of the ultrasound system was fixed over the patellar tendon as described above. Tendon elongation was recorded during five isometric ramp contraction trials, which were performed from rest to 90% of the MVC within ~ 5 s. The participants received visual feedback of the target and generated moments during the ramp contractions. We recorded five ramp trials in order to achieve a high reliability of tendon elongation measurements^35^. The elongation of the patellar tendon was measured as described above and the rest length was measured in the resting state prior to the contraction at full relaxation and equilibrium of moments. The measured moments were corrected for axis misalignments between the knee and dynamometer axes and for gravitational forces using inverse dynamics^36^. For this purpose, reflective markers were placed at the anatomical reference points trochanter major, condylus lateralis femoris, condylus medialis femoris, and the medial and lateral malleolus, and the kinematic data was recorded with eight cameras of a Vicon motion capture system (version 2.1, Vicon Motion Systems, Oxford, UK) at 250 Hz. To calculate the knee extension moments, the contribution of antagonistic coactivation to the resultant knee joint moment was assessed based on the relationship of the antagonistic muscle (i.e., biceps femoris) EMG-activity and the corresponding knee flexion moment using the procedure suggested by Mademli et al.^34^. The force on the patellar tendon was then calculated by dividing the knee extension moments by the lever arm of the tendon, which was predicted based on anthropometry^25^ and adjusted to the corresponding knee angle during contraction^37^. Maximum tendon force (TF_max_) was determined based on the maximum knee extension moment generated during the MVC trials of each participant. To calculate the patellar tendon force–elongation relationship from each participant, all measured data from the five single ramp trials were used. First, the patellar tendon force–elongation relationship for each single ramp trial was saved as a piecewise-linear polynomial function in MATLAB (version R2019b; ‘interp1’ function with ‘pp’ option), using the measured data points (pairs of tendon force and tendon elongation values) of each ramp as input and defining a linear function between two subsequent measured data points. Using the defined ramp specific linear functions, we calculated tendon force and tendon elongation for every data point of each ramp and the five tendon force–elongation data points were averaged. We used a linear fit in the average force–elongation data points between 50 and 90% of the maximum tendon force to calculate patellar tendon stiffness. The normalized patellar tendon stiffness was calculated as product of the stiffness and the rest length. To map the operating patellar tendon strains measured during the squats onto the force-strain curve achieved during the MVCs, the individual average patellar tendon force-strain data points were fit with a second-order polynomial passing through zero. For displaying the average of all participants’ force-strain relationship the means of the coefficients of the individual polynomial functions were calculated.

### Estimation of patellar tendon strain during loaded back squats

To estimate patellar tendon strain during the squats, maximum strain during the MVC and the percentage of 1RM were used as explanatory variables. The measured average patellar tendon strain around the maximum knee flexion during the squats (i.e., ~50° to 60° during the flexion and extension phase) at 40%, 60% and 80% of the 1RM loading conditions was set as dependent variable. Using the method of least squares we derived the parameters of the logarithmic function

1 $$f\left(x,y\right)=\left({a}_{1}x+{a}_{2}\right)\left(\mathrm{ln}\left({a}_{3}y-{a}_{4}\right)+{a}_{5}\right)$$

where *x* denotes the percentage of the 1RM, *y* the maximum strain during the isometric MVC and ${a}_{1}$,…,${a}_{5}$ the unknown coefficients. To support the legitimation of the fitting function, the linearity between 40%, 60% and 80% of 1RM load and operating strain during squats was calculated for each participant*.* Logarithmic regression fits for the relationship between the strain during an MVC and the strain at each 1RM percentage during squats were conducted separately.

### Statistics

The statistical analyses were performed using R v4.0.1 (R foundation for statistical computing, Vienna, Austria). A linear mixed-effects model (LMM) was used (nlme package) to test the main effect of the factor load (40%, 60%, 80% of 1RM) on the investigated parameters (i.e. patellar tendon elongation, resultant knee joint moment, resultant hip joint moment, resultant ankle joint moment, patellar tendon strain, normalized EMG-activity, resultant hip to knee joint moment ratio, resultant ankle to knee joint moment ratio and trunk angle). If a significant main effect was found, post-hoc analyses were performed between factor levels (emmeans package), and Benjamini–Hochberg corrected p-values taking care for false discovery rate (FDR) are reported. Normality assumption was checked for the normalized residuals using the Shapiro–Wilk test and was not violated in any parameter. For the calculation of the average tendon elongation during squats, three recordings were included in 30% of the cases, while two recordings were used in the remaining 70% due to artifacts in the ultrasound images. The relationship between the percentage of the 1RM and tendon strain during the squat was calculated individually for each participant with a linear regression. The relationship between tendon strain during the MVC and strain during the squats at each percentage of the 1RM was separately calculated with a logarithmic regression. It is to mention, that this analysis was not preplanned and was conducted subsequent to the observation that the logarithmic fit was most appropriate to fit the available data. The reproducibility of the measured patellar tendon strain in each 1RM percentage of the squats was determined using the intraclass correlation coefficients of the investigated trials (ICC; two-way mixed, average measurement, absolute agreement) and was defined as poor (ICC < 0.5), moderate (ICC from 0.5 to 0.75), good (ICC from 0.75 to 0.9) and excellent (ICC > 0.9) according to the suggestions by Koo and Li^38^. Correlations between outcome parameters were calculated using the Pearson correlation coefficient (*r*). A paired *t*-test was used to compare the measured tendon rest length between measurement days. The significance level was set to α = 0.05. Cosine similarity (CS) was used to assess the similarity of the vertical GRF-patterns between dominant and non-dominant leg in the movement range specified above.

2 $$CS=\mathrm{cos}(\theta )= \frac{A\cdot B}{\Vert A\Vert \cdot \Vert B\Vert }=\frac{\sum_{i=1}^{n}{A}_{i}{B}_{i}}{\sqrt{\sum_{i=1}^{n}{A}_{i}^{2}}\sqrt{\sum_{i=1}^{n}{B}_{i}^{2}}}$$

*A* represents the vector of the vertical GRF of the dominant leg and *B* the vector of the GRF of the non-dominant leg. The values of CS range from 1, meaning exactly the same pattern, to − 1, meaning exactly the opposite. Furthermore, for the GRF we conducted statistical parametric mapping (SPM) two-tailed paired t-tests with Bonferroni-corrected significance t* level for multiple comparisons between each relevant pair of levels (i.e. dominant and non-dominant leg at 40, 60 and 80% of 1RM). Similar to the previously described analyses, the significance level was set to α = 0.05 before adjustment. SPM calculations were performed using the open-source package spm1d (v. 0.4.3 for MATLAB version R2019b).

## Results

The participants’ 1RM load, knee joint moment, patellar tendon force, patellar tendon elongation and patellar tendon strain during the MVC as well as normalized patellar tendon stiffness, patellar tendon lever arm and rest length are presented in Table 1. We did not find a significant difference (*p* = 0.350) in tendon rest length measured between the squat and MVC sessions. There was a high correlation between the 1RM load and the maximum knee extension moment during the MVC (*r* = 0.98, *p* < 0.001). Further, we found a significant relationship (*r* = 0.75, *p* = 0.012) between the maximum force applied to the patellar tendon during the MVC and normalized patellar tendon stiffness.

**Table 1:** One repetition maximum load during the squats, knee joint moment, force applied to the patellar tendon, patellar tendon elongation and strain during the isometric maximum voluntary knee extension contraction (MVC) as well as patellar tendon stiffness, normalized patellar tendon stiffness, lever arm and rest length measured in the two measurement sessions, (*n* = 10, Mean ± SD).

Parameter	
One repetition maximum (kg)	208 ± 68
Knee joint moment during MVC (Nm)	326 ± 104
Tendon force during MVC (N)	5142 ± 1361
Tendon elongation during MVC (mm)	4.7 ± 0.9
Tendon strain during MVC (%)	10.30 ± 1.95
Tendon stiffness (N/mm)	1474 ± 665
Normalized tendon stiffness (kN/strain)	68.05 ± 31.20
Tendon lever arm (mm)	62.8 ± 5.2
Tendon rest length in the squat session (mm)	45.9 ± 5.1
Tendon rest length in the MVC session (mm)	45.7 ± 5.0

**Figure 2:**
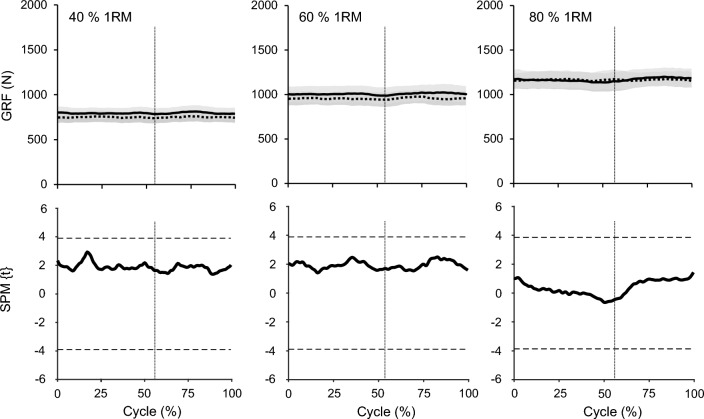
Vertical ground reaction forces (GRF) of the dominant leg (solid line) and non-dominant leg (dotted line) over the 10° of flexion and 10° of extension to/from the reversal point at 60° (separated with vertical dotted line) at 40%, 60% and 80% of the one repetition maximum (1RM) are shown in the top row as means ± standard error (shaded area). The paired *t*-test statistic continuum SPM{t} over the movement cycle for each load level between legs is shown below. The critical threshold t* =  ± 3.89 (dashed line) for α = 0.05 represents the null hypothesis that is rejected when SPM{t} exceeds this threshold. No statistically significant effect was found between the dominant and non-dominant leg at any percentage of the 1RM.

**Figure 3:**
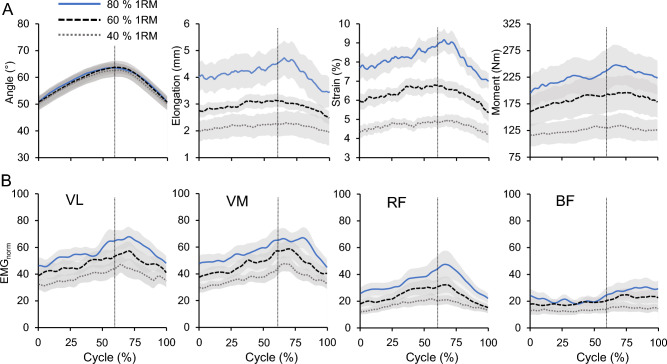
(**A**) Knee joint angle, patellar tendon elongation, patellar tendon strain and resultant knee joint moment during squats at 40%, 60% and 80% of the one repetition maximum (1RM). The cycle (%) describes the time-normalized squat movement with 10° flexion and 10° extension to/from the reversal point (separated with dotted line). (**B**) EMG-activity normalized to maximum voluntary isometric contractions (EMG_norm_) of the vastus lateralis (VL), vastus medialis (VM), rectus femoris (RF) and biceps femoris (BF). Mean ± standard error (shaded areas).

**Figure 4:**
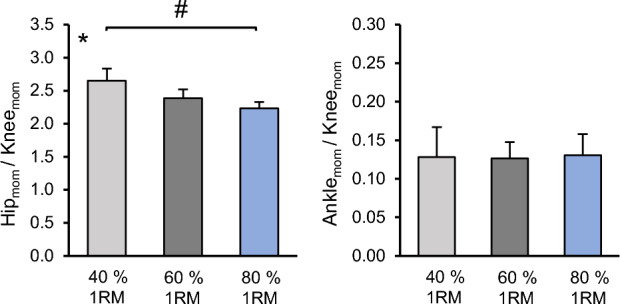
Mean and standard error of mean of the resultant hip to knee joint moment (left) and ankle to knee joint moment ratio (right). *Significant main effect of % 1RM load, *p* < 0.001, ^#^Bracket indicates significant post hoc comparisons.

**Figure 5:**
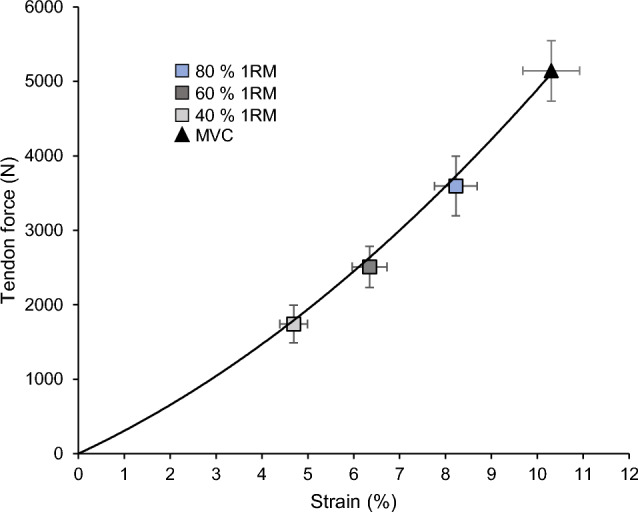
Operating patellar tendon strain during squats at 40%, 60% and 80% of the one repetition maximum (1RM) mapped onto the patellar tendon force–strain relationship that was experimentally determined during a maximum isometric voluntary knee extension contraction (MVC). The solid line represents the mean second-order polynomial. Mean ± standard error.

The CS of the vertical GRF between the dominant and non-dominant leg were very high in all percentages of 1RM (*CS* = 0.99 ± 0.01). Further, the SPM analysis revealed no statistically significant differences between the two legs for each level (t* = 3.89, *p* > 0.05; Fig. 2). The analyzed range of motion of the knee joint angle and the respective patellar tendon elongation, patellar tendon strain, resultant knee joint moments and EMG-activity of VL, VM, RF and BF during the squats are depicted in Fig. 3. There was a statistically significant effect of load on the average patellar tendon elongation (*p* < 0.001), resultant knee joint moment (*p* < 0.001), resultant hip joint moment (*p* < 0.001), resultant ankle joint moment (*p* < 0.001), patellar tendon strain (*p* < 0.001), trunk angle (*p* < 0.001) and normalized EMG-activity (VL, VM, RF *p* < 0.001; BF *p* = 0.040) of the four investigated muscles (Table 2), as well as on the resultant hip to knee joint moment ratio (*p* < 0.001; Fig. 4). The ankle to knee joint moment ratio did not show a significant load effect (Fig. 4). The post hoc comparisons demonstrated a continuous increase (*p* < 0.05) of the resultant joint moments, average patellar tendon elongation/strain and muscles EMG-activity with the percentage of 1RM load (Table 2). The comparison of the hip to knee joint ratio (Fig. 4) showed a significant decrease from 40% 1RM to 80% 1RM (*p* = 0.018) but no significant difference between 60% 1RM and 80% 1RM (*p* = 0.051) or 40% 1RM and 60% 1RM (*p* = 0.100). Further, we found a significantly greater trunk angle at 80% 1RM compared to 40% 1RM (*p* = 0.020), yet there was no significant difference between 80% 1RM and 60% 1RM (*p* = 0.223) and between 60% 1RM and 40% 1RM (*p* = 0.090). We found excellent intraclass correlations of the measured patellar tendon strain during squats of 0.919 at 40% 1RM, 0.924 at 60% 1RM and 0.922 at 80% 1RM. We did not find significant relationships between the 1RM load and patellar tendon strain during the squats (*r* = − 0.12 to *r* = − 0.24, *p* = 0.499 to *p* = 0.750). Figure 5 shows the operating patellar tendon strain values of the 1RM percentages in squats mapped onto the experimentally determined patellar tendon force-strain relationship during the isometric knee extension contractions.

**Table 2:** Average patellar tendon elongation and strain, resultant knee, hip and ankle joint moment, trunk angle and electromyographic activity (EMG) normalized to maximum voluntary isometric contractions of the vastus lateralis (VL), vastus medialis (VM), rectus femoris (RF) and biceps femoris (BF) during squats with 40%, 60% and 80% of the one repetition maximum (1RM; *n* = 10, Mean ± SD).

Parameter	40% 1RM	60% 1RM	80% 1RM
Tendon elongation (mm)*	2.4 ± 1.4 a	2.9 ± 0.7 b	4.2 ± 1.9 c
Tendon strain (%)*	4.67 ± 0.95 a	6.35 ± 1.19 b	8.22 ± 1.46 c
Knee joint moment (Nm)*	126 ± 68 a	184 ± 102 b	226 ± 106 c
Hip joint moment (Nm)*	307 ± 123 a	409 ± 176 b	486 ± 191 c
Ankle joint moment (Nm)*	7 ± 20 a	25 ± 28 b	33 ± 31 c
Trunk angle (°)*	68 ± 4 a	70 ± 5 a,b	71 ± 5 b
VL-EMG (%)*	38.7 ± 17.3 a	48.1 ± 18.2 b	56.8 ± 18.6 c
VM-EMG (%)*	37.3 ± 12.6 a	47.3 ± 17.0 b	56.9 ± 20.3 c
RF-EMG (%)*	17.5 ± 9.6 a	24.6 ± 13.3 b	34.7 ± 19.1 c
BF-EMG (%)*	13.9 ± 9.3 a	20.1 ± 12.5 b	23.7 ± 14.2 c

*Significant effect of load. Different letters within a row indicate significant differences between means (*p* < 0.05, post-hoc analysis).

**Figure 6:**
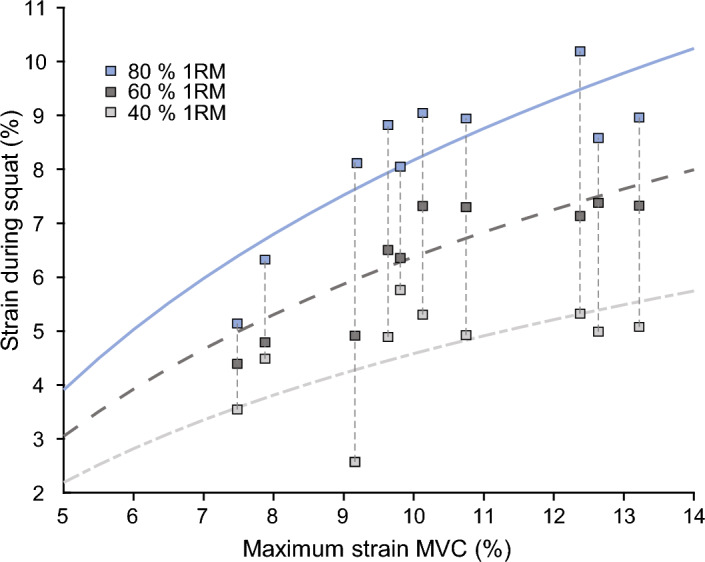
Logarithmic relationship between the maximum patellar tendon strain during the isometric maximum voluntary knee extension contraction (MVC) and the average strain values during the squats at 40%, 60% and 80% of one repetition maximum (1RM). The vertical dotted lines connect the measured strain values of each participant.

**Figure 7:**
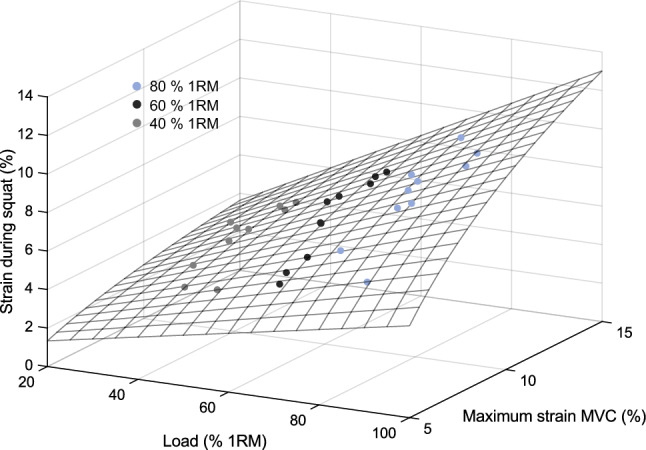
Estimation of patellar tendon strain during squats (surface area) by using the percentage of the one repetition maximum (1RM) and the maximum patellar tendon strain during the maximum isometric contraction (MVC) as explanatory variables. The spheres present the measured individual values of the participants at the different load levels, respectively.

We found a curvilinear (logarithmic) relationship between the average patellar tendon strain during the squats and the maximum patellar tendon strain during the MVC (40% 1RM: R^2^ = 0.286, 60% 1RM: R^2^ = 0.781, 80% 1RM: R^2^ = 0.697; Fig. 6). While there was a strong linear relationship between patellar tendon strain and the percentage of 1RM load within the participants (R^2^ = 0.970 ± 0.041), the inter-individual variability of the achieved strain values during the squats was high (Fig. 6). The individual patellar tendon strain ranged from 2.6 to 5.8% at 40% 1RM, from 4.4 to 7.4% at 60% 1RM and from 5.1 to 10.2% at 80% 1RM. Further, there was a significant (*p* < 0.05) negative relationship between the maximum patellar tendon strain during the MVC and the normalized EMG-activity of the knee extensors during the loaded squats (VL: *r* = − 0.66, − 0.75, − 0.75; VM: *r* = − 0.84, − 0.82, − 0.76; RF: *r* = − 0.60, − 0.73, − 0.67 for 40, 60 and 80% of 1RM, respectively). The agreement between the measured and estimated patellar tendon strain during squats using Eq. (1) with the coefficients (${a}_{1}$= 0.0676, ${a}_{2}$= 0.7496, ${a}_{3}$= 710.615, ${a}_{4}$= − 1.0, ${a}_{5}$= − 7.5405) was high (R^2^ = 0.858, Fig. 7).

## Discussion

We investigated patellar tendon strain during squats around the optimum joint angle for knee extension moment generation (i.e. 60° knee joint angle) at 40%, 60% and 80% of 1RM to quantify tendon strain, as strain expresses the internal mechanical demand of the tissue and is the crucial mechanical stimulus for tendon adaptation^3,4,13^. Mechanical loading of tendons inducing strains between 4.5 and 6.5% provides an effective training stimulus to improve tendon mechanical and morphological properties^11–13^. The average patellar tendon strain values were between 4.7 and 8.2% during the investigated squats, which shows that the mechanical loading during medium-weight squats (40% to 60% of 1RM) might already be sufficient to promote anabolic patellar tendon responses. However, the individual variability of the achieved strains during the different percentages of the 1RM was too high to generalize these findings, which confirms our first hypothesis. Yet, it was possible to estimate the individual patellar tendon strain during squats as a function of the maximum patellar tendon strain during an isometric MVC and the 1RM percentages with high accuracy (R^2^ = 0.858), which also confirms our second hypothesis.

The resultant knee joint moments during the squats ranged on average from 126 to 226 Nm, which agrees with earlier reported values^20,21^. There was a progressive increase in the resultant knee, hip and ankle joint moment as well as in the EMG-activity of the investigated knee extensor muscles with increasing percentages of the 1RM in all participants, indicating a gradual increase of muscle loading. Looking at the average patellar tendon strain values it seems that the target strain of 4.5–6.5% may be achieved between 40 and 60% of the 1RM. However, with this recommendation five participants would be overloaded (strain > 6.5%) and two underloaded (strain < 4.5%). This is 70% of the examined participants and, thus, too large for individual training recommendations. The variability in tendon strain observed in our study may be attributed to several factors. First, individual differences in squat execution may contribute to this variability. The comparison of the ground reaction force curves between the dominant and non-dominant leg showed high similarity and no significant differences, suggesting a quite symmetrical execution of the squats. Thus, we can argue that the load distribution between the two legs had no relevant effect on the individual variability in tendon strain. Second, the relative contributions of the resultant moments within the lower extremity joints may affect the measured strain during the squats. We found a shift towards a more upright trunk position as load increased, which resulted in a decreased contribution of the hip joint relative to the knee joint with increasing load indicating a potential effect on the found tendon strain variability. Finally, potential imbalances between muscle strength and tendon stiffness within the investigated participants could also contribute to the observed variability in tendon strain. A high individual fluctuation in the patellar tendon strain achieved during isometric knee extension MVCs has been reported in several earlier studies^24,25,27^ despite the general association between knee extensor muscle strength and normalized patellar tendon stiffness^24^. In the current study, we also found a significant relationship between maximum force applied to the patellar tendon during the MVC and normalized patellar tendon stiffness, nevertheless, the maximum achieved strain ranged from 7.5 to 13.2% (Fig. 6), evidencing imbalances between muscle strength and tendon stiffness at the individual level. The lower rate of tendon remodeling compared to muscle^39^, the tissue-specific effective mechanical stimuli for tendon and muscle adaptation^11,13^ and possible individual biological disposition^40^ may influence the balance between muscle strength and normalized tendon stiffness. An imbalance between muscle strength and normalized tendon stiffness can result in different tendon strain values at a given percentage of the MVC or 1RM between individuals. Due to the high variability in patellar tendon strain at a given 1RM percentage, it is barely possible to predict the individual patellar tendon strain based on the 1RM. The association between the 1RM load and the achieved patellar tendon strain during the squats was low (on average R^2^ = 0.198) and, therefore, the current recommendations for load prescriptions in training and clinical practice^23^ are unsuitable to personalize tendon loading.

Tendon loading causes an increase of both synthesis and degradation of tendon matrix components^7,41^. There are indications for a certain range or ‘sweet spot’ of tendon strain that triggers a net anabolic response and promotes the adaptation of the tendon’s mechanical and morphological properties, while too high or too low levels of strain may be associated with impairments of tendon structure^7,9,10^. Repetitive loading of the tendon with a strain magnitude between 4.5 and 6.5% has been shown to be an effective mechanical stimulus, improving tendon properties^11–13^. In some of our investigated athletes, this target strain range could be achieved at 40% 1RM, in others only at 60% or 80% 1RM, which clearly demonstrates the limitations of a personalized regulation of tendon loading based on the 1RM. We found a strong relationship between the maximum knee joint moment during the isometric MVC and the 1RM, indicating that the participants with higher MVCs also show a greater 1RM and, consequently, higher average resultant knee joint moments at a given 1RM percentage during the squat. This relationship suggests a potential individual association between patellar tendon strain during the MVC and patellar tendon strain during squats.

A curvilinear (logarithmic) relationship between tendon strain during the squats and maximum tendon strain during the MVC was found. Based on this curvilinear relationship and the almost linear relationship of the achieved individual strain values between the 1RM percentages, it was possible to estimate tendon strain during squats as a function of the maximum patellar tendon strain during the MVC and the percentage of 1RM loading. The agreement between the estimated and measured strain values during the squats was high (R^2^ = 0.858). Therefore, this approach may provide new opportunities for individualized patellar tendon loading recommendations in clinical and sports practice. Patellar tendon properties during an isometric MVC can be measured quite easily and the determination of maximum strain is considerably less challenging compared to tendon strain measurements during a dynamic squat. It may therefore be possible to individually predict the target patellar tendon strain with the presented estimation function and to prescribe exercise loads that correspond to the ‘sweet spot’ region for tendon anabolic responses during squats. However, it should be mentioned that, in the current study, the reported estimation coefficients have not been cross-validated (i.e., with data from participants which were not part of the used dataset) and, therefore, the accuracy of a prediction needs to be verified.

The found logarithmic relationship between tendon strain during the squats and maximum tendon strain during the MVC indicates a lower ratio of the two variables with increased maximum tendon strain during the isometric MVC. Thus, individuals that demonstrate high levels of patellar tendon strain during isometric contractions do not show equally increased levels of patellar tendon strain during dynamic squats. We found a negative association between maximum patellar tendon strain during the isometric MVC and the normalized EMG-activity of the knee extensor muscles during the squats, indicating a reduction of muscle activation in the participants with high tendon strain values. The decreased EMG-activity of the knee extensors during squats with increasing maximum patellar strain during the MVC may imply a neural inhibition to reduce the mechanical demand on the tendon and might partly explain the observed curvilinear relationship between maximum patellar tendon strain during the isometric MVC and the squats.

In summary, our results show a high linear relationship between the 1RM load and patellar tendon strain during loaded back squats at the individual level. However, the variability between the investigated participants was too high to regulate patellar tendon strain during squats only based on the 1RM. We were able to estimate the individual patellar tendon strain during back squats as a function of the more easily measurable maximum patellar tendon strain during an isometric MVC and the percentage of 1RM load in squats with high accuracy.

## Perspectives

Tendon strain magnitude is a key regulatory factor for tendon adaptation and there are indications for a ‘sweet spot’ of strain magnitude that facilitates tendon adaptation. As the actual tendon strain during exercise cannot be predicted based on the 1RM, load prescription in training and clinical settings should be rethought, particularly with the progression of new opportunities for personalized tendon loading. The prediction of individual tendon strain magnitude during exercise loading using easily measurable explanatory variables may introduce new possibilities in personalized decision-making for tendon loading. In this context, our findings for the patellar tendon in back squats may give some new research impulses towards the development of individual tendon loading predictions in a wide range of functional exercises.

## Data Availability

The data that support the findings of this study are available from the corresponding author upon reasonable request.
